# Increased Neutrophil Secretion Induced by *NLRP3* Mutation Links the Inflammasome to Azurophilic Granule Exocytosis

**DOI:** 10.3389/fcimb.2017.00507

**Published:** 2017-12-11

**Authors:** Jennifer L. Johnson, Mahalakshmi Ramadass, Ariela Haimovich, Matthew D. McGeough, Jinzhong Zhang, Hal M. Hoffman, Sergio D. Catz

**Affiliations:** ^1^Department of Molecular Medicine, The Scripps Research Institute, La Jolla, CA, United States; ^2^Division of Pediatric Allergy, Immunology, and Rheumatology, Rady Children's Hospital of San Diego, University of California, San Diego, La Jolla, CA, United States

**Keywords:** inflammasome, exocytosis, neutrophil, inflammation, azurophilic granule, interleukin-1, innate immunity, Toll-like receptor 9

## Abstract

Heterozygous mutations in the *NLRP3* gene in patients with cryopyrin associated periodic syndrome (CAPS) lead to hyper-responsive inflammasome function. CAPS is a systemic auto-inflammatory syndrome characterized by the activation of the innate immune system induced by elevated pro-inflammatory cytokines, but the involvement of selective innate immune cells in this process is not fully understood. Neutrophil secretion and the toxic components of their granules are mediators of inflammation associated with several human diseases and inflammatory conditions. Here, using the *Nlrp3*^*A350V*^ inducible mouse model (*MWS CreT*) that recapitulates human patients with the A352V mutation in *NLRP3* observed in the Muckle-Wells sub-phenotype of CAPS, we studied the relationship between hyper-activation of the inflammasome and neutrophil exocytosis. Using a flow cytometry approach, we show that *Nlrp3*^*A350V*^ (MWS) neutrophils express normal basal levels of CD11b at the plasma membrane and that the upregulation of CD11b from secretory vesicles in response to several plasma membrane or endocytic agonist including the bacterial-derived mimetic peptide formyl-Leu-Met-Phe (fMLF) and the unmethylated oligonucleotide CpG is normal in MWS neutrophils. Significant but modest CD11b upregulation in MWS neutrophils compared to wild type was only observed in response to GM-CSF and CpG. The same pattern was observed for the secretion of matrix metalloproteinase-9 (MMP-9) from gelatinase granules in that MMP-9 secretion in MWS neutrophils was not different from that observed in wild-type neutrophils except when stimulated with GM-CSF and CpG. In contrast, azurophilic granule secretion, whose cargoes constitute the most toxic secretory and pro-inflammatory factors of the neutrophil, was markedly dysregulated in MWS neutrophils under both basal and stimulated conditions. This could not be attributed to paracrine effects of secretory cytokines because IL-1β secretion by neutrophils was undetectable under these experimental conditions. The increased azurophilic granule exocytosis in MWS neutrophils was attenuated by treatment with the neutrophil exocytosis inhibitor Nexinhib20. In agreement with a possible neutrophil contribution to systemic inflammation in CAPS, the levels of neutrophil secretory proteins were significantly elevated in the plasma from *Nlrp3*^*A350V*^ mice. Altogether, our data indicates an azurophilic granule-selective dysregulation of neutrophil exocytosis in CAPS.

## Introduction

Systemic inflammatory syndromes caused by either genetic defects, exacerbated innate immune responses to infection, trauma or autoimmune disorders are characterized by elevated plasma levels of pro-inflammatory cytokines and by the activation of the innate immune system, but the specific cellular mediators of systemic inflammation are not fully identified, and the mechanisms of neutrophil-mediated systemic inflammation are not well understood.

Neutrophils, and in particular their secretory proteins, are important mediators of systemic inflammation. Uncontrolled neutrophil activation has been proposed to play a significant role in the development of sepsis syndrome, and timely neutrophil depletion has been proposed as a plausible therapy (Brown et al., 2006). Neutrophil secretory proteins have also been associated with the development of systemic inflammatory response syndrome (Johnson et al., 2004), thrombohemorrhagic vasculitis (Hirahashi et al., 2006), fibrotic disorders (Gregory et al., 2015), ischemia-reperfusion injury (Matthijsen et al., 2007; Shimoda et al., 2007), and endothelial dysfunction (Eiserich et al., 2002), and are critical effectors in the development of toxic shock (Tkalcevic et al., 2000).

Neutrophil secretory organelles are distinguished by their different cargo content and their distinct tendency to undergo exocytosis which correlates with the roles of these cargoes in the sequential neutrophil functions in infection and inflammation (Borregaard and Cowland, 1997; Ramadass and Catz, 2016). During systemic inflammation, circulating neutrophils are capable of releasing granule contents from their most toxic (azurophilic) granules into the blood stream. Increased plasma levels of myeloperoxidase and the serine protease, elastase, are hallmarks of neutrophil activation in circulation and are often seen in sepsis and endotoxemia, as well as in animal models of these syndromes (Tanaka et al., 1991; Johnson et al., 2011; Kothari et al., 2011). Myeloperoxidase is a pro-oxidant molecule largely associated with the development of cardiovascular disease (Nicholls and Hazen, 2005) and endothelial malfunction (Eiserich et al., 2002), a common feature of systemic inflammation. Elevated neutrophil elastase plasma levels are also associated with pro-inflammatory syndromes including fibrosis and vasculitis, while cathepsin G mediates inflammation-induced tissue injury after ischemia-reperfusion in kidneys, and both neutrophil elastase- and cathepsin G-mediated inflammation and thrombospondin-1 degradation facilitate cancer progression (El Rayes et al., 2015).

Azurophilic granule exocytosis is regulated by the small GTPase Rab27a and its effector JFC1 (Munafo et al., 2007; Brzezinska et al., 2008; Johnson et al., 2010a,b). Small-molecules that interfere with the binding of JFC1 with Rab27a impaired granule docking, fusion and release in neutrophils (Johnson et al., 2016). The importance of neutrophil exocytosis in systemic inflammation is highlighted by our recent work showing that treatment of endotoxemic mice with the neutrophil exocytosis inhibitors (Nexinhibs) decreases plasma myeloperoxidase levels and reduces neutrophil tissue infiltration (Johnson et al., 2016).

The inflammasome is a multi-protein complex, expressed primarily in myeloid cells, which is responsible for the processing and secretion of IL-1β and IL-18 (Martinon et al., 2002; Strowig et al., 2012). Mature IL-1β production requires priming signals to induce transcription of pro-IL-1β and pro-IL-18 and NLRP3 inflammasome components, and a second signal to initiate inflammasome assembly and activation leading to the activation and cleavage of caspase-1 (Martinon et al., 2002). Activated caspase-1, in turn, cleaves the immature pro-cytokines to their active forms (IL-1β and IL-18) that are then released from the cell (Petrilli et al., 2007). The NLRP3 inflammasome has been implicated in numerous common inflammatory disorders (e.g., gout and atherosclerosis) (Martinon et al., 2006; Duewell et al., 2010) as well as normal and dysregulated host response to infection (e.g., Streptococcus and malaria) (Harder et al., 2009; Shio et al., 2009).

Heterozygous mutations in the *NLRP3* gene observed in patients with cryopyrin associated periodic syndrome (CAPS) lead to a hyper-responsive inflammasome that does not require a second signal for activation (Hoffman et al., 2001; Hoffman and Broderick, 2016). Humans and mice with these gain-of-function mutations present with recurrent or chronic systemic inflammatory symptoms involving the skin, musculoskeletal system, and central nervous system (Brydges et al., 2009). Neutrophilic infiltration in several tissues is a hallmark of CAPS but the regulatory roles of the NLRP3 inflammasome on neutrophil function, particularly granule release, is poorly understood.

Here, we show for the first time that neutrophils from a mouse model of CAPS display a selective exocytosis disorder manifested as exacerbated azurophilic granule cargo release even under basal conditions, both *ex vivo* and *in vivo*. Our data highlights a novel mechanism of neutrophil exocytosis regulation and highlights neutrophil exocytosis as a feature of inflammasome dysregulation.

## Materials and methods

### Animal model of caps

Our experiments utilize the *Nlrp3*^*A350V*^ inducible mouse model (*MWS CreT*) that is homologous to human CAPS patients with the Muckle-Wells A352V mutation in *NLRP3*. The tamoxifen-inducible *Nlrp3*^A350V/+^ cre *ERT2* and control animals were injected i.p. with 50 mg/kg tamoxifen free base for 4 days as previously described(McGeough et al., 2012). The mice (6–12 weeks old) had access to food and water *ad libitum*. All animal studies were performed in compliance with the U.S. Department of Health and Human Services Guide for the Care and Use of Laboratory Animals and according to NIH and institutional guidelines. All animal protocols and procedures were approved by the University of California San Diego Institutional Animal Care and Use Committees (IACUC).

### Materials

The stimuli used for treating cells were: GM-CSF (Shenandoah, 200-15), CpG ODN 1826 (InvivoGen, tlrl-1826), CL097 (InvivoGen, tlrl-c97), fMLF (Sigma), PMA (Sigma). The antibodies used for flow cytometry staining are as follows: FITC-conjugated anti-Ly6G (clone 1A8, BD biosciences) and Alexa Fluor 647-conjugated anti-mouse CD11b (clone M1/70, BD biosciences). The anti-Nlrp3 antibody used in this study was from R&D (MAB7578), and detects both human and mouse proteins. Nexinhib20, 4,4-dimethyl-1-(3-nitrophenyl)-2-(1*H*-1,2,4-triazol-1-yl)pent-1-en-3-one was described previously, its molecular signature and purity were confirmed by mass spectrometry analysis (Johnson et al., 2016). Nexinhib20 was dissolved at a concentration of 10 mM in DMSO and diluted in PBS. The final concentration of Nexinhib20 in the reaction media was 10 μM.

### Mouse neutrophil isolation

Bone marrow-derived neutrophils were isolated using a Percoll gradient fractionation system as described (Johnson et al., 2012). A three-layer Percoll gradient was used (52, 64, and 72%) and neutrophils were isolated from the 64 to 72% interface, washed, and used in the assays. The purity of the neutrophil population isolated by Percoll sedimentation after bone marrow filtration was 80–89% as determined by Ly6G^+^/CD11b^+^ double-positive staining. This is now shown in Supplementary Figure S1.

### Neutrophil stimulation

Purified mouse neutrophils (1 × 10^6^) were resuspended in phenol red-free RPMI and either stimulated with GM-CSF (10 ng/ml) or left untreated for 30 min at 37°C at which point the cells were stimulated with either 5 μM CpG ODN 1826 for 1 h, 10 μg/ml of CL097 for 1 h, 1 μM fMLF for 10 min or 100 ng/ml PMA for 30 min at 37°C as described (Ramadass et al., 2016). Where indicated, neutrophils were incubated with the neutrophil exocytosis inhibitor Nexinhib20 (Johnson et al., 2016) for 1 h before the addition of stimuli. Following the treatments, the cells were spun down at 800 g for 10 min, the cell-free supernatant was collected for ELISA analysis and the cells were stained for flow cytometry analysis.

### Flow cytometry

To stain for cell-surface markers, cells were blocked in ice-cold PBS containing 1% BSA, and stained with fluorescein isothiocyanate (FITC)-conjugated anti-Ly6G (clone 1A8, BD biosciences) and Alexa Fluor 647-conjugated anti-mouse CD11b (clone M1/70, BD biosciences). The cells were then washed and fixed in 1.5% paraformaldehyde in PBS. The samples were analyzed using a BD LSR II flow cytometer (BD Biosciences) and the data was analyzed using FlowJo software.

### Enzyme-linked immunosorbent assay (ELISA)

Matrix metalloproteinase-9 (MMP-9) levels in the supernatant were assessed using the R&D Mouse Pro-MMP-9 ELISA kit (DY909) as per the manufacturer's protocol. Myeloperoxidase (MPO) levels in the supernatant were assessed using the R&D Mouse Myeloperoxidase DuoSet ELISA (DY3667) as per the manufacturer's protocol. For *in vivo* studies of myeloperoxidase and matrix-metalloproteinase 9 plasma levels, blood samples were collected by cheek bleeding in K3EDTA MiniCollect tubes (Greiner Bio-One, Austria) and processed for myeloperoxidase and MMP-9 determination by ELISA as described above.

### Phagocytosis assay

Neutrophils (1 × 10^6^) were incubated in the presence of 5 × 10^6^ serum-opsonized TexasRed-zymosan particles. The samples were spun down to increased contact between particles and neutrophils and incubated for 30 min at 37°C. The neutrophils were transferred to coverslips and fixed with 3.7% paraformaldehyde for 8 min. The samples were then incubated in 1% BSA, 0.01% saponin, PBS for 1 h for blocking and subsequently incubated with the primary antibody anti-mouse MPO for 12 h at 4°C. Endogenous MPO was detected using alexa-488-conjugated secondary antibodies and the samples were analyzed by confocal microscopy as described previously (Monfregola et al., 2012; He et al., 2016; Johnson et al., 2016). Samples were analyzed with a Zeiss LSM 710 laser scanning confocal microscope attached to a Zeiss Observer Z1 microscope at 21°C, using a 63 × oil Plan Apo, 1.4 numerical aperture (NA) objective. Images were collected using ZEN-LSM software and processed using ImageJ (National Institutes of Health, Bethesda, MD) and Photoshop CS4 (Adobe). Analysis of colocalization was performed using ZEN software.

### Statistical analysis

Data are presented as means, and error bars correspond to standard errors of the means (SEMs). Statistical significance was determined using the unpaired or paired Student's *t*-test using GraphPad Prism (version 6). For comparison of multiple groups, one-way ANOVA followed by Fisher's LSD *post hoc* test was performed. All graphs were made using GraphPad Prism (version 6) software. Grubb's test was used to determine statistical outliers.

## Results

Although neutrophils are likely to be important mediators of systemic inflammation in CAPS, the mechanisms underlying neutrophilic infiltration and putative neutrophil activation associated with mutations in *NLRP3* (which codes for cryopyrin) are poorly understood. Here, we chose to study neutrophils from the *Nlrp3*^*A350V*^ inducible mouse model (*MWS CreT*) that models human patients with the A352V mutation in *NLRP3* observed in the Muckle-Wells sub-phenotype of CAPS. Mice with constitutive expression of this mutation live <2 weeks making it difficult to study adequate numbers of inflammatory cells (Brydges et al., 2009). The tamoxifen inducible Cre ERT2 and conditional *Nlrp3*^A350V^ mutation of the *MWS CreT* mice allow for *in vivo* or *ex vivo* induction of the mutation with tamoxifen in an adult mouse.

### The effect of the Nlrp3^A350V^ (MWS) mutation and inflammasome activation on CD11b expression in neutrophils

**Figure 1:**
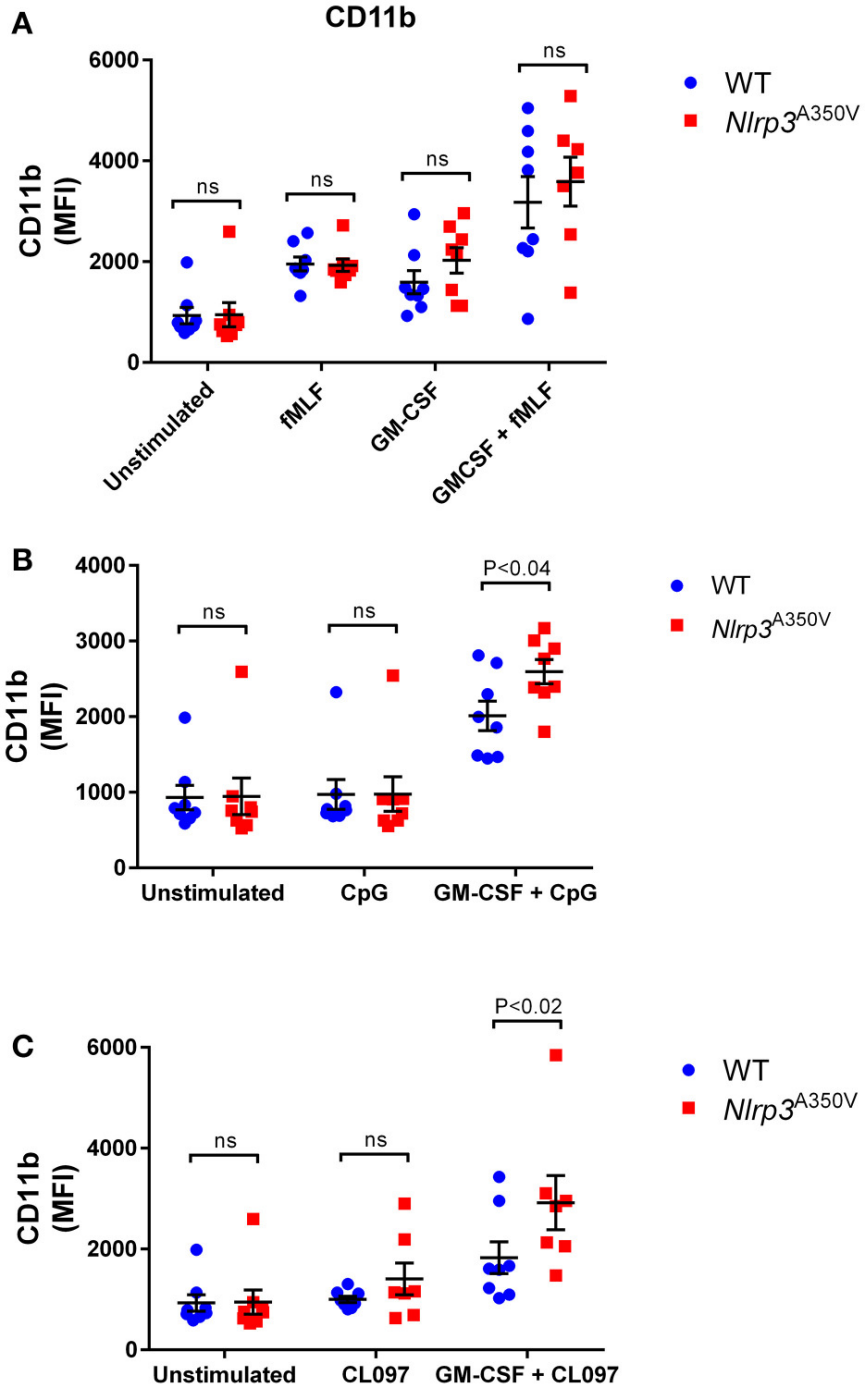
Effect of the Nlrp3^A350V^ mutation on secretory vesicle exocytosis in neutrophils. **(A–C)**, The mobilization of secretory vesicles was analyzed by monitoring the upregulation of the neutrophil adhesion molecule CD11b at the plasma membrane by flow cytometry using specific antibodies that detect extracellular epitopes of the indicated marker. Wild type (WT: blue) or MWS neutrophils (Nlrp3 mutant: red) were treated with GM-CSF or vehicle for 30 min and subsequently stimulated with the indicated agonists as described under “Materials and Methods.” Each symbol corresponds to an individual mouse and the error bars indicate mean ± SEM of 8 independent mice from 3 independent experiments. Significant differences between groups were calculated using ANOVA followed by Fisher's *post-hoc* test. ns, not significant.

CD11b is a subunit of Mac1 (CD11b/CD18), a β2 integrin that regulates tissue infiltration and mediates neutrophil adhesion to fibrinogen (Wright et al., 1988), intercellular adhesion molecule-1 (Diamond et al., 1990) and collagen (Walzog et al., 1995). In mouse neutrophils this protein is stored mainly in neutrophil secretory vesicles and is upregulated at the plasma membrane upon stimulation. Here, isolated neutrophils were double-labeled with antibodies specific for the neutrophil marker Ly6G and for CD11b (Supplementary Figure S1), and Ly6G^+^ neutrophils were analyzed for the expression of CD11b by flow cytometry. We show that *Nlrp3*^*A350V*^ (MWS) neutrophils express normal basal levels of CD11b at the plasma membrane (Figure 1A). Furthermore, the upregulation of CD11b at the plasma membrane in response to the bacterial-derived mimetic peptide formyl-Leu-Met-Phe (fMLF) is not impaired in the mutant neutrophils as these cells show a 2-fold increase in CD11b expression similar to that of wild type controls (Figure 1A, *P* < 0.02 for both wild type and MWS neutrophils, unstimulated *vs* fMLF). GM-CSF is a pluripotent cytokine that is markedly elevated in MWS mice (Brydges et al., 2013) and is a known sensitizer (primer) of neutrophil responsiveness to second stimuli (Ramadass et al., 2016). Wild type and MWS-neutrophils show similar levels of CD11b at the plasma membrane when stimulated with GM-CSF (Figure 1A). Furthermore, no differences were observed between wild type and mutant neutrophils when CD11b plasma levels were measured upon sequential stimulation with GM-CSF and fMLF (Figure 1A), suggesting again that the chemotactic peptide signals through intracellular pathways that are independent of NLRP3.

Next, we analyzed the responsiveness of CD11b-positive vesicles of wild type and MWS-neutrophils to ligands of the nucleic-acid-sensing endocytic Toll-like receptor (TLR)-7 and TLR-9 pathways in both resting and GM-CSF-sensitized cells. The inclusion of the double agent stimulation scheme with GM-CSF plus one of the endocytic TLR agonists, CpG-oligodeoxynucleotide (CpG, TLR9 agonist) or CL097 (TLR7 agonist), is justified by the following reasons: (a) The NLRP3 inflammasome is known to sense multiple types of endocytic TLR nucleic acids (Sha et al., 2014); (b) GM-CSF is elevated in *Nlrp3*^A350V^ and is thought to play a significant role in systemic inflammation caused by the inflammasome activation (Brydges et al., 2013) and (c) the GM-CSF-endocytic TLR ligand combination was recently recognized by our group as a strong stimuli of neutrophil exocytosis (Ramadass et al., 2016). As shown previously by our group (Ramadass et al., 2016), CD11b upregulation in response to endocytic TLR ligands is enhanced in GM-CSF-primed neutrophils in wild type cells (Figures 1B,**C**, *P* < 0.001 and *P* < 0.03 for CpG and CL097 *vs* unstimulated control, respectively). MWS-neutrophils also showed enhanced CD11b upregulation after GM-CSF priming (Figures 1B,**C**, *P* < 0.0001 for both CpG and CL097 *vs* unstimulated control, respectively). Significant differences in CD11b upregulation between wild type and *Nlrp3*^A350V^ cells were observed in response to both the TLR9 agonist CpG and the TLR7 ligand CL097 under GM-CSF-primed conditions (Figure 1B,C, respectively), suggesting that both the inflammasome activation and GM-CSF sensitize the endocytic signaling pathway that controls secretory vesicle exocytosis in neutrophils.

### Endocytic TLR-specific upregulation of gelatinase granule exocytosis in inflammasome activation

**Figure 2:**
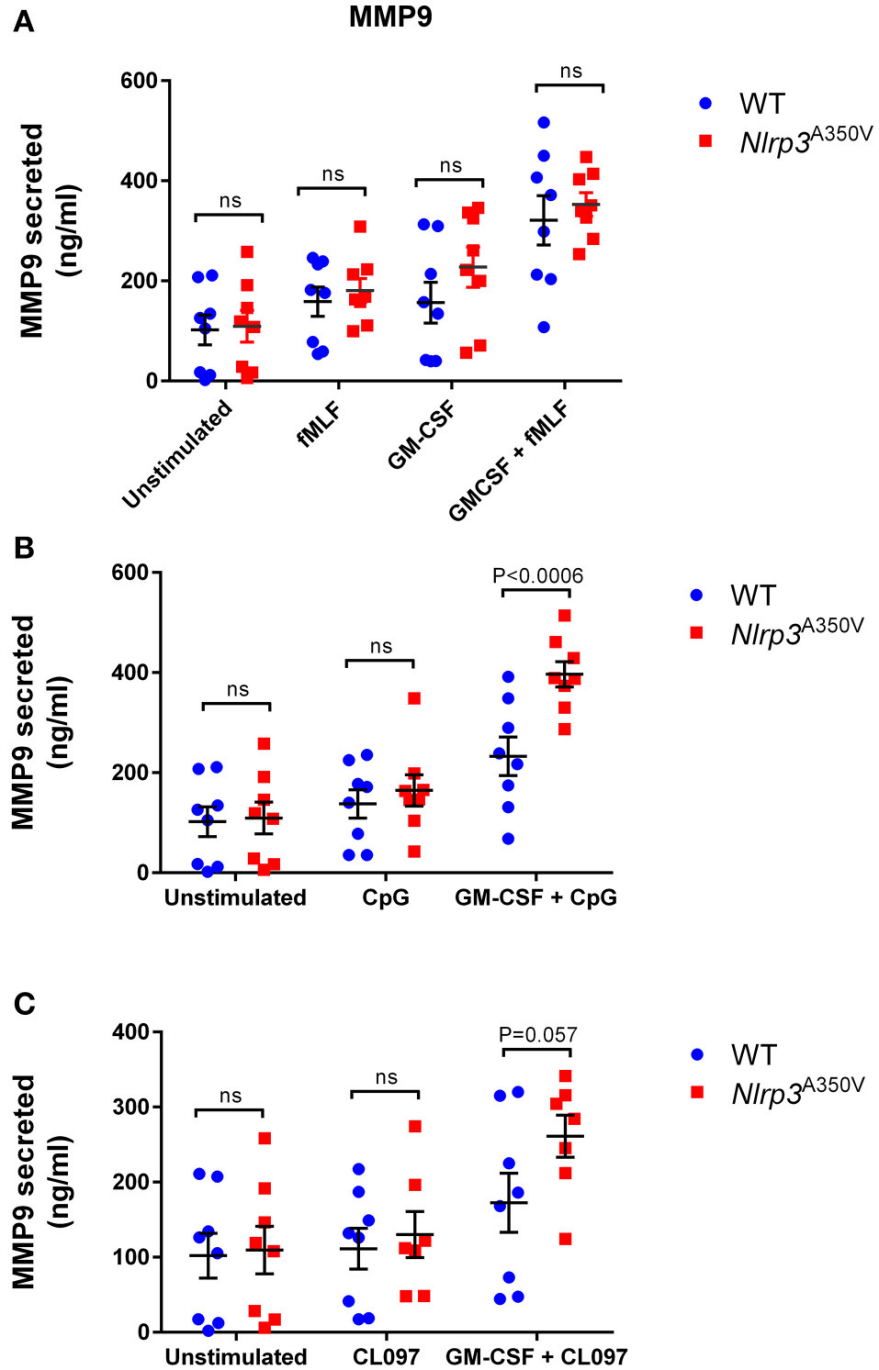
Effect of the Nlrp3^A350V^ mutation on gelatinase granule exocytosis in neutrophils. **(A–C)**, The mobilization of gelatinase granules was analyzed by monitoring the secretion of gelatinase B (MMP-9) by ELISA as described under “Materials and Methods.” Wild type (WT: blue) or MWS neutrophils (Nlrp3 mutant: red) were treated with GM-CSF or vehicle for 30 min and subsequently stimulated with the indicated agonists as described under “Materials and Methods.” Each symbol corresponds to an individual mouse and the error bars indicate mean ± SEM of 8 independent mice from 3 independent experiments. Significant differences between groups were calculated using ANOVA followed by Fisher's *post-hoc* test. ns, not significant.

Tissue-infiltrating neutrophils are the major source of the pro-inflammatory MMP-9 enzyme (gelatinase B), which is known to not only facilitate angiogenesis and the progression of inflammation by promoting the release of extracellular matrix-bound or cell-surface-bound cytokines, such as VEGF (Bergers et al., 2000), but also to process IL-1β precursors into its active form (Schonbeck et al., 1998). Here, we study the mobilization of the MMP-9-containing gelatinase granules in neutrophils expressing the MWS mutation under basal, stimulated and primed conditions (Figure 2). Similar to wild type cells (Ramadass et al., 2016), MWS-neutrophils responded efficiently to fMLF stimulation by secreting MMP-9 (Figure 2A, *P* < 0.0004 and *P* < 0.006, for wild type and MWS cells, respectively, unstimulated *vs* fMLF, paired Student's *t-test*). In response to GM-CSF, the secretion of MMP-9 was not significantly different between wild type and MWS cells, thus establishing that neutrophils with the MWS *Nlrp3*^A350V^ mutation are not particularly sensitive to GM-CSF stimulation (Figure 2A). In addition, no differences were observed in MMP-9 secretion between wild type and MWS neutrophils in response to GM-CSF-priming and subsequent fMLF-stimulation (Figure 2A, *P* < 0.0001, unstimulated vs. GM-CSF + fMLF for both wild type and MWS mutant cells), suggesting that the amplified secretory response of gelatinase granules to fMLF is independent of any putative interference induced by the inflammasome activation in MWS-neutrophils. Similar to that observed for CD11b, gelatinase granule exocytosis was significantly elevated in *Nlrp3*^A350V^ neutrophils in response to the TLR9-specific agonist CpG-ODN (Figure 2B) and moderately increased in response to the TLR7 agonist CL097 (Figure 2C) under GM-CSF-primed conditions, highlighting a possible TLR-specific NLRP3-dependent pathway in MWS-neutrophils when activated by the endocytic TLR agonists, in particular through TLR9.

### Dysregulated activation of azurophilic granule exocytosis induced by inflammasome upregulation

**Figure 3:**
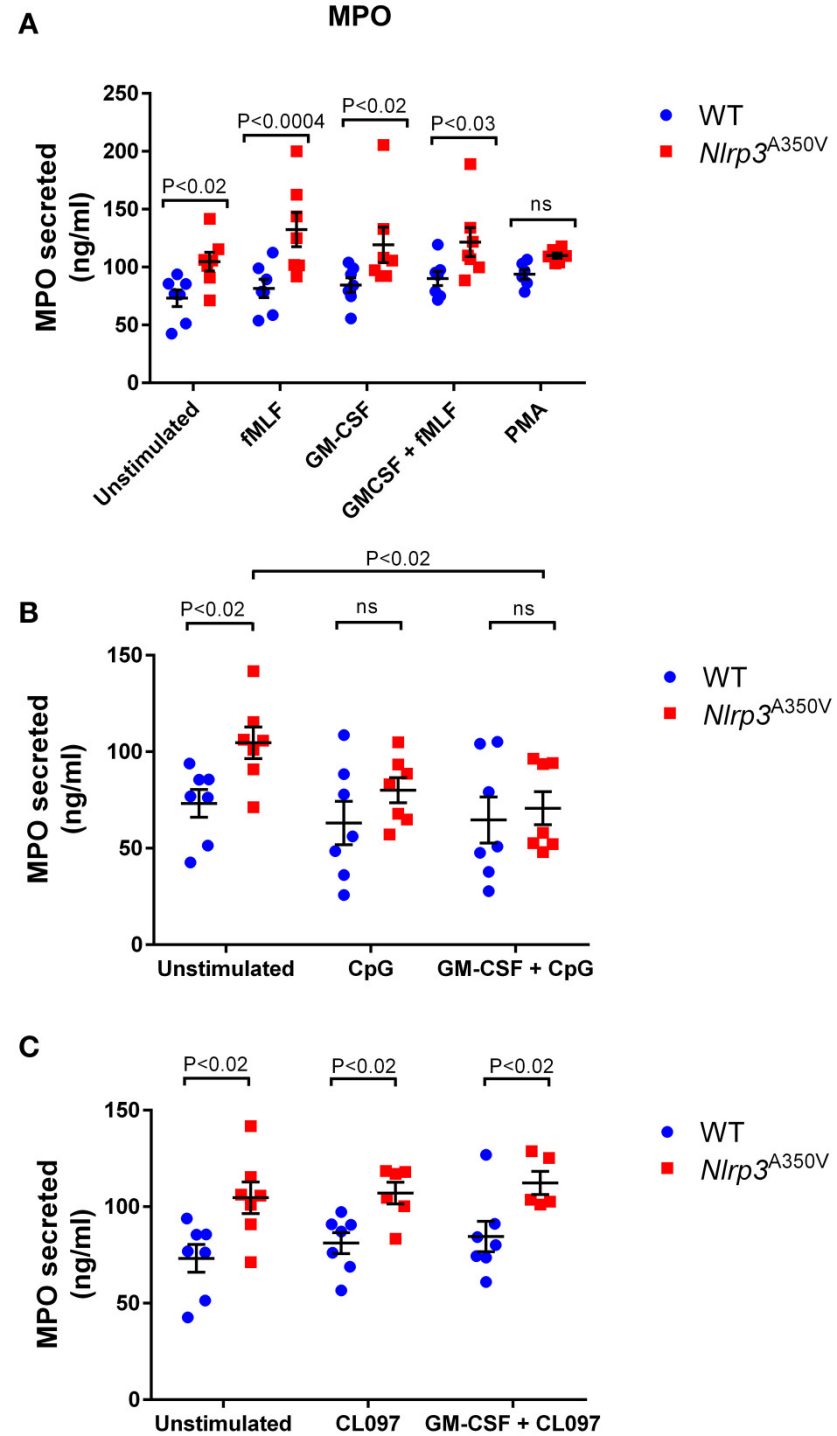
Effect of the Nlrp3^A350V^ mutation on azurophilic granule exocytosis in neutrophils. The mobilization of azurophilic granules was analyzed by monitoring the secretion of myeloperoxidase (MPO) by ELISA as described under “Materials and Methods.” **(A–C)** Wild type (WT; blue) or MWS neutrophils (Nlrp3 mutant; red) were treated with GM-CSF or vehicle for 30 min and subsequently stimulated with the indicated agonists as described under “Materials and Methods.” Each symbol corresponds to an individual mouse and the error bars indicate mean ± SEM of 7 independent mice from 3 independent experiments. Significant differences between groups were calculated using ANOVA followed by Fisher's *post-hoc* test. ns, not significant.

Azurophilic granule cargoes constitute the most toxic secretory and pro-inflammatory factors of the neutrophil. Unregulated secretion of this granule subset, including myeloperoxidase, elastase, and cathepsin G, leads to inflammatory processes associated with human disease. Here, we show for the first time that neutrophils with the MWS *Nlrp3*^A350V^ mutation present a dysregulated secretion of azurophilic granule exocytosis (Figure 3). Using myeloperoxidase as a cargo marker, we show that azurophilic granule exocytosis is dramatically increased under basal conditions in MWS-mutant neutrophils with activated inflammasome (Figure 3A). This significantly enhanced exocytosis of this granule subset is maintained under stimulated conditions. For most stimuli, the levels of azurophilic granule exocytosis activity in MWS-neutrophils are not higher than those observed under basal conditions, with the exception of fMLF-stimulated MWS-mutant neutrophils (Figure 3A, *P* < 0.05) indicating that the inflammasome activation leads to unregulated secretion of azurophilic granule cargoes to levels similar to those observed under stimulated conditions. These data indicate that the MWS *Nlrp3*^A350V^ mutation induces azurophilic granule exocytosis to an extent that most likely involves the full-reservoir of secretory azurophilic granules. Increased azurophilic granule exocytosis in MWS-neutrophils was not observed under CpG-stimulated conditions (Figure 3B) but was significantly increased under CL097 stimulation (Figure 3C). Altogether, these results suggest the presence of an intrinsic defective mechanism of azurophilic granule exocytosis induced by the inflammasome activation in MWS. Importantly, IL-1β was undetectable in the supernatants of wild type and MWS neutrophils both under stimulated or unstimulated conditions (data not shown). Thus, the increased azurophilic granule cargo secretion in MWS-neutrophils is independent of a putative paracrine action of IL-1β in the incubation media and is not induced by direct stimulation or priming effect induced by this cytokine.

### The delivery of azurophilic granule content to the phagosome is not affected in Nlrp3^A350V^ mutant neutrophils

**Figure 4:**
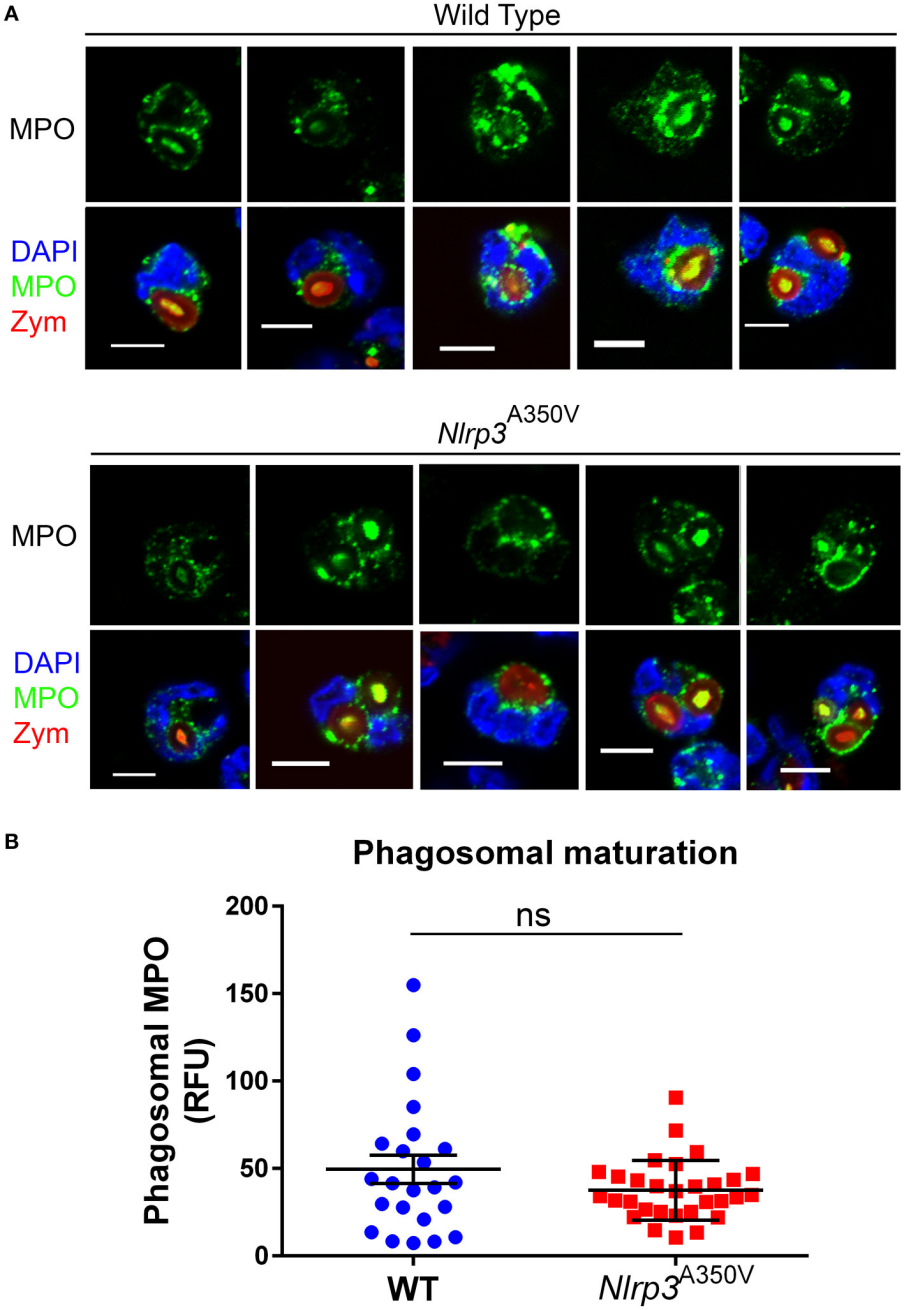
The delivery of azurophilic granule cargo to the phagosome is not affected in Nlrp3^A350V^ MWS neutrophils. Isolated neutrophils were incubated in the presence of serum-opsonized Texas-Red zymosan for 30 min, fixed and analyzed for the distribution of endogenous myeloperoxidase (green) by immunofluorescence microscopy. **(A)**, Representative images of five different phagocyting wild type and MWS mutant neutrophils are shown. **(B)**, Quantitative analysis of the relative fluorescence intensity surrounding each phagosome from 23 phagosomes from wild type cells and 30 phagosomes from MWS mutant cells. The bars represent the mean ± SEM. No significant differences between these two groups were observed (ns), using unpaired Student's *t*-test.

To study whether the increased recruitment of azurophilic granules was a plasma membrane exclusive phenomena or was also observed in phagolysosomes of *Nlrp3*^A350V^ neutrophils, wild type and MWS neutrophils were incubated in the presence of opsonized zymosan and phagocytosis was analyzed by confocal microscopy. Both, wild type and *Nlrp3*^A350V^ neutrophils phagocytosed only one particle with only 22 and 18% of neutrophils phagocyting more than one zymosan particle, respectively. Zymosan phagosomes were surrounded by MPO-positive staining in both wild type and MWS mutant neutrophils (examples of wild type or *Nlrp3*^A350V^ neutrophils phagocyting cells are shown in Figure 4A). Quantitative analysis of phagosomal maturation calculated as the fluorescence intensity of phagosomal endogenous MPO showed no significant differences between wild type and MWS mutant neutrophils (Figure 4B). These data suggest that the effect of NLRP3 activation is selective for exocytosis, rather than a general increase in mobilization of azurophil granules.

### Elevated neutrophil secretory proteins in plasma of Nlrp3^A350V^ mutant mice

**Figure 5:**
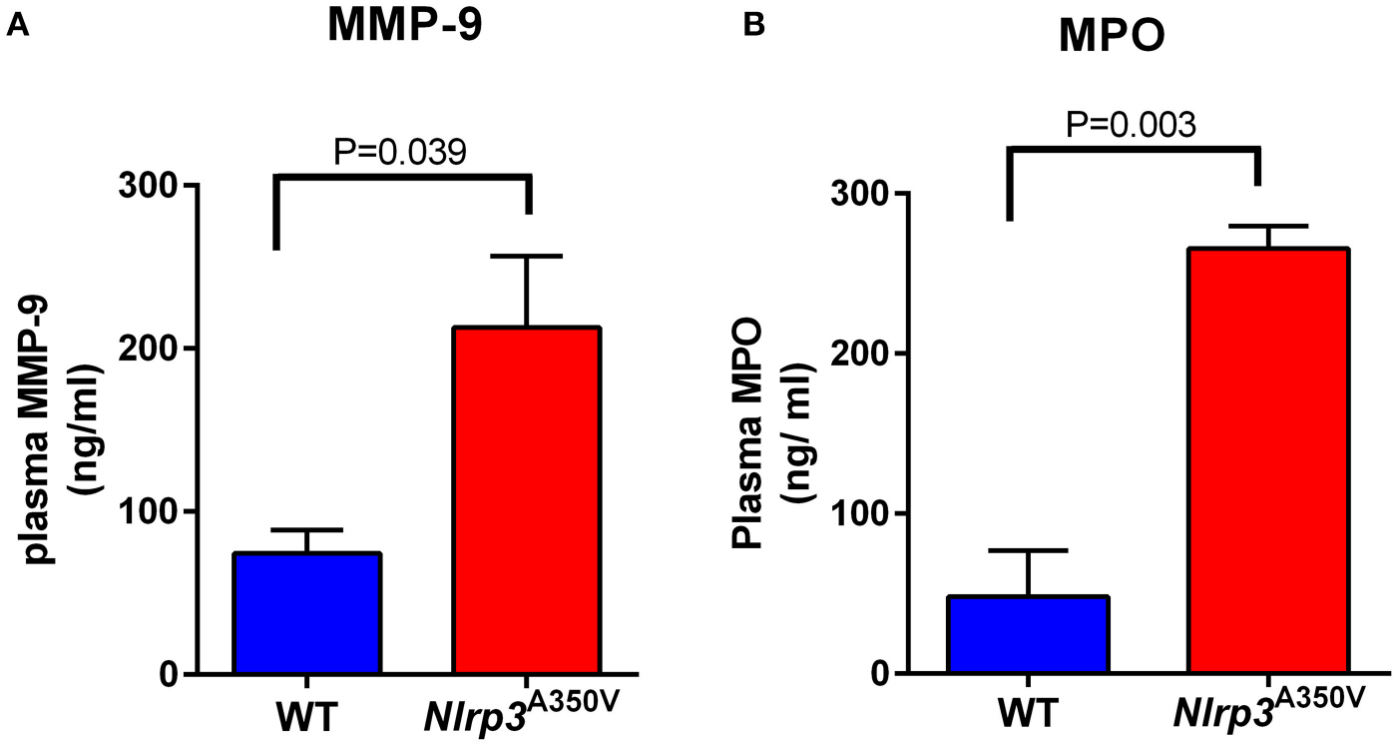
The Nlrp3^A350V^ MWS mice show increased *in vivo* gelatinase and azurophilic granule exocytosis. The plasma levels of the gelatinase and azurophilic granule cargoes, gelatinase B (MMP-9) **(A)** and myeloperoxidase (MPO) **(B)**, respectively, were analyzed by ELISA in wild type (WT) and MWS (Nlrp3 mutant) mice as described under “Materials and Methods.” The data is presented as mean ± SEM and is representative of 3 independent experiments each with 3–5 mice per group. Significant differences between WT and mutant mice were calculated using unpaired Student's *t*-test.

To analyze whether neutrophil activation takes place in MWS *in vivo*, we next analyzed the levels of neutrophil secretory proteins in plasma from wild type and MWS mice. In Figure 5 we show that MWS-mice has a 3-fold increase in plasma levels of the secretory protein MMP-9 (Figure 5A) and a 5-fold elevation in circulating levels of MPO (Figure 5B). The levels of secretory proteins in plasma were measured under basal conditions. The results mimic those observed in normal mice after challenge with pro-inflammatory molecules, for example, lipopolysaccharide (Johnson et al., 2011), suggesting that in MWS disease, neutrophils are activated in circulation, a phenotype that contributes to the marked pro-inflammatory state of MWS mice. These data also correlate with previous studies from our laboratory showing that MWS mice are characterized by increased levels of pro-inflammatory cytokines in circulation, including IL-1β, IL-18, GM-CSF, IFNγ, TNFα, and IL-6 (Brydges et al., 2013). Of note, the results obtained with uninduced (no tamoxifen treatment) *Nlrp3*^A350V/+^ cre *ERT2* controls were comparable to those obtained with wild type controls of matching background. For example, we show that the differences in the levels of plasma MPO between uninduced and tamoxifen-induced *Nlrp3*^A350V^ cre ERT2 mice are similar to those observed with wild type mice (Supplementary Figure S2A). Similar results were observed for MMP-9 secretion which was normal (wild type levels) in uninduced mice (Supplementary Figure S2B).

### Inhibitors of neutrophil exocytosis decrease azurophilic granule exocytosis in Nlrp3^A350V^ neutrophils

**Figure 6:**
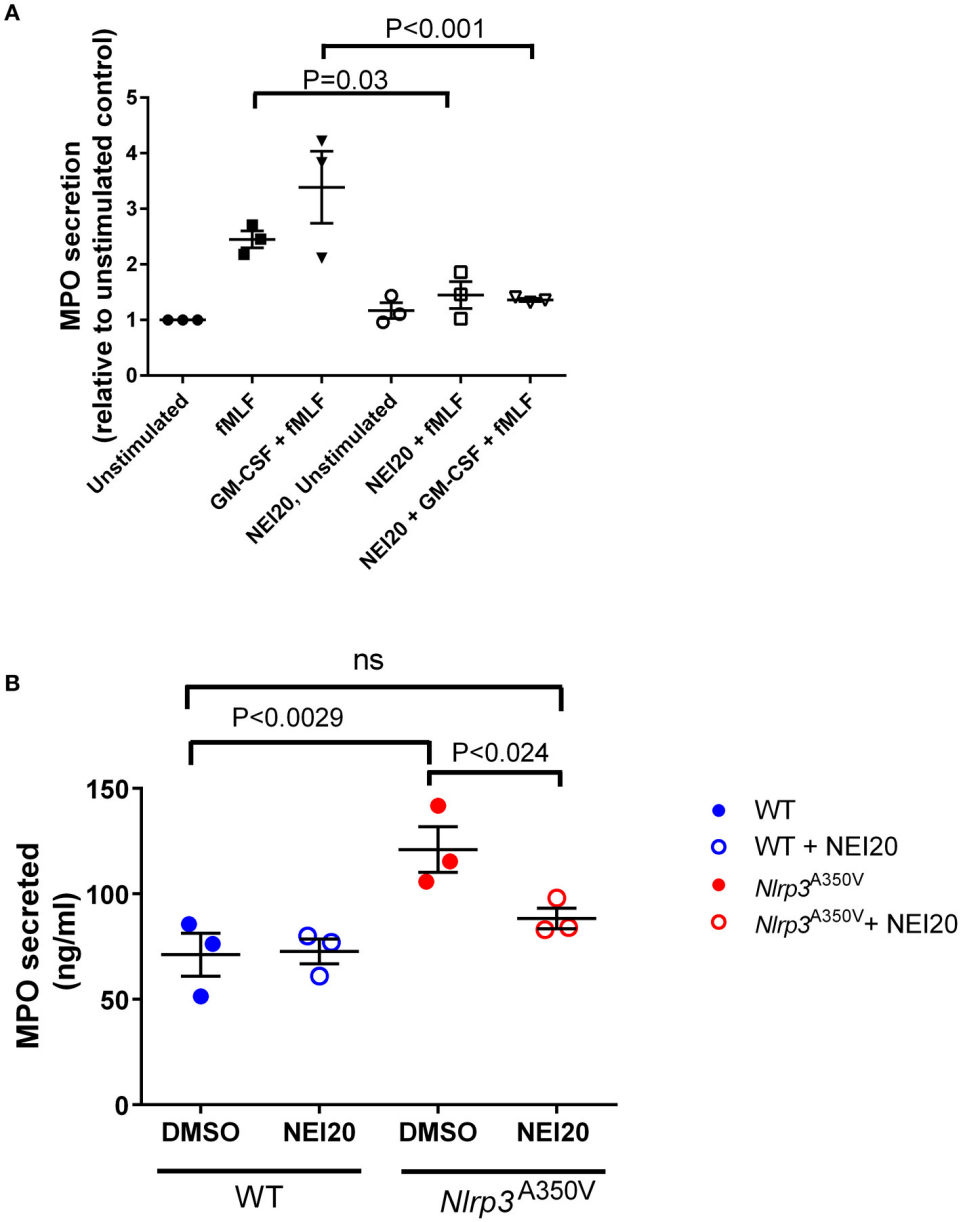
Functional analyses identify Nexinhib20 as an efficient inhibitor of azurophilic granule exocytosis in Nlrp3^A350V^ MWS neutrophils. The mobilization of azurophilic granules by wild type or *Nlrp3*^A350V^ MWS neutrophils was analyzed by monitoring the secretion of myeloperoxidase (MPO) by ELISA as described under “Materials and Methods.” **(A)** Wild type neutrophils were incubated in the presence of Nexinhib20 (NEI20) at 10 μM or DMSO and subsequently stimulated with fMLF, GM-CSF + fMLF or left unstimulated. **(B)** Unstimulated wild type or Nlrp3A350V MWS neutrophils were treated with Nexinhib20 and MPO secretion was determined as above. The data is represented as mean ± SEM (*n* = 3). Significant differences between groups were calculated using ANOVA followed by Fisher's *post-hoc* test. ns, not significant.

In a recent work, we identified and characterized the first class of neutrophil exocytosis inhibitors (Johnson et al., 2016). These small-molecules inhibit neutrophil exocytosis by interference with the binding of the small GTPase Rab27a, a master regulator of exocytosis, with its native effector JFC1 (synaptotagmin-like protein 1, Slp1) (Johnson et al., 2016). These compounds named Nexinhibs, have the ability to decrease inflammation by inhibiting azurophilic granule exocytosis. Because of their pharmacological and clinical implications, we next analyzed whether treatment with the most potent Nexinhib (NEI20) affects exocytosis of neutrophils with the MWS *Nlrp3*^A350V^ mutation. First, we analyzed the effect of NEI20 on GM-CSF-primed or unprimed fMLF-stimulated neutrophil exocytosis in the wild type mice, and show that myeloperoxidase secretion is significantly inhibited by the inhibitor Nexinhib20 in mouse neutrophils (Figure 6A). Next, we show that *in vitro* treatment with 10 μM Nexinhib20 for 1 h reduces the basal activation of MWS-neutrophils azurophilic granule exocytosis to wild type levels (Figure 6B). Altogether, our data suggest that neutrophil exocytosis inhibitors may be potentially useful for the treatment of neutrophil-induced systemic inflammation in cryopyrin-associated periodic syndromes.

## Discussion

Neutrophil exocytosis and the release of their toxic cargoes is a characteristic of systemic inflammation. Patients with cryopyrin-associated periodic syndromes and the corresponding mouse models present with mild increase in white blood cell counts but pronounced neutrophilia and tissue neutrophilic infiltration (Brydges et al., 2013). Here we show for the first time that neutrophils with the MWS *Nlrp3*^A350V^ mutation, a mouse model of the human disease Muckle-Wells syndrome, are characterized by unrestricted azurophilic granule exocytosis, thus linking inflammasome activation with neutrophil secretion-mediated inflammation. Although secretory vesicles and gelatinase granules of neutrophils expressing the MWS *Nlrp3*^A350V^ mutation show mild but significantly elevated secretion in response to selective stimuli including GM-CSF with the TLR9 ligand CpG, unstimulated secretion was not observed for these set of granules, and their secretory responses to stimuli was largely normal in MWS neutrophils. Instead, the secretion of the most toxic cargoes from azurophilic granules of MWS neutrophils was characterized by unrestricted activation, with MWS neutrophils showing high exocytosis parameters even under unstimulated conditions. Marked elevated levels of azurophilic granule secretory proteins were also observed *in vivo* in the MWS mouse model suggesting that neutrophil exocytosis is an important component of the systemic inflammatory response induced by inflammasome activation associated with cryopyrin-associated periodic syndromes. It is worth noting that GM-CSF + CpG is the only condition for which MPO detection in the supernatant is lower than the control basal levels in mutant cells. We attribute this phenomenon to two possible scenarios: (a) The significant effect of GM-CSF + CpG on the upregulation of CD11b, a MPO binding protein (Lau et al., 2005), in MWS cells (Figure 1B), possibly facilitates CD11b-mediated recruitment of some of the secreted MPO to the plasma membrane and thus reduces the detectable MPO pool in the supernatant. (b) It is possible that CpG induces endocytosis of MPO. This is however less likely as it would require a selective mechanism (MMP-9 is not reduced after CpG treatment).

Comparative analysis of the secretion profiles of gelatinase and azurophilic granules *ex vivo* and *in vivo* indicates a general dysregulation of azurophilic granule exocytosis in MWS neutrophils but a selective *in vivo* activation of gelatinase secretion in these mutant cells. Thus, while gelatinase secretion is highly upregulated in circulation in MWS mice, isolated neutrophils with the MWS *Nlrp3*^A350V^ mutation show only a mild increase in gelatinase exocytosis when stimulated, and do not show elevated basal levels of gelatinase in the supernatants when incubated in the absence of stimuli. These data suggest that the increased levels of gelatinase observed in the plasma of MWS mice are due to an exacerbated response induced by the elevated levels of circulatory cytokine that act as priming agents. In particular, GM-CSF, a pro-inflammatory cytokines that is elevated in the plasma of MWS mice (Brydges et al., 2013), has been recently shown by our group to prime neutrophils to increase gelatinase granule exocytosis (Ramadass et al., 2016). However, the marked increase in upregulation of azurophilic granules was observed not only *in vivo*, but most notably *in vitro*, under experimental conditions that (a) do not induce azurophilic granule exocytosis in wild type cells and (b) do not induce cytokine secretion. Altogether, these data highlight an intracellular mechanism induced by the inflammasome activation that triggers the deregulation of azurophilic granule exocytosis. However, this is not mediated by differences in Nlrp3 expression as we did not observe significant differences in NLRP3 protein levels between wild type and MWS mutant mice (Supplementary Figure S3).

Neutrophil exocytosis and in particular azurophilic granule cargo secretion is regulated by the small GTPase Rab27a and its effector molecule JFC1 (Munafo et al., 2007; Brzezinska et al., 2008; Johnson et al., 2012). In a previous work, we identified Nexinhib20, the first neutrophil exocytosis inhibitor, a small-molecule that specifically interferes with the binding of Rab27a to JFC1 and prevents both azurophilic granule exocytosis and *in vivo* neutrophil inflammation in a model of systemic inflammation induced by LPS (Johnson et al., 2016). Here we show that Nexinhib20 inhibits the exacerbated azurophilic granule exocytosis observed in MWS neutrophils indicating that NLRP3 activation induces exocytosis through the activation of Rab27a and its effector JFC1. The mechanisms of cross-regulation of these two pathways is unknown and under current investigation in our lab. One possibility is that the inflammasome activation induces actin depolymerization to facilitate exocytosis. Interestingly a mechanism of NLRP3 inflammasome regulation by F-actin via Flightless-I (FliI), a member of the gelsolin superfamily of actin-remodeling proteins, and LRRFIP2, a leucine-rich repeat protein that interacts with FliI, has recently been described (Burger et al., 2016) although whether the NLRP3 inflammasome counter regulates actin polymerization is currently unknown. Independently of the role of actin on the dysregulation of azurophilic granule exocytosis in MWS mutant mice, based on the observation that neutrophil exocytosis inhibitors downregulate exocytosis of *Nlrp3*^A350V^ neutrophils, it is tempting to speculate that these compounds could help decrease neutrophil-dependent pro-inflammatory functions *in vivo*, both in animal models and in patients with MWS. However, because of the pleiotropic effects of the multiple inflammatory mediators upregulated in the disease model (Brydges et al., 2013), it is likely that neutrophil inhibitors would be more effective in combined therapies with cytokine inhibitors.

Altogether, we present the first study of neutrophil exocytosis in inflammasome activation and identified a selective mechanism of azurophilic granule exocytosis activation caused by NLRP3 inflammasome upregulation. As these granules contain the most toxic cargoes of neutrophil storage compartments, and exocytosis dysregulation occurs both *in vitro* and *in vivo*, our study suggest that increased neutrophil secretory proteins contributes to systemic inflammation in CAPS.

## Author contributions

JJ and MR performed experiments, analyzed data, contributed ideas and comments and contributed to manuscript writing; AH and MM performed experiments, analyzed data and contributed ideas and comments; HH, designed experiments, contributed reagents, ideas and comments; JZ, performed experiments and analyzed data, SC conceived the idea, designed the manuscript and the experimental approach, analyzed data and wrote the manuscript with input from JJ, MR, MM, and HH.

### Conflict of interest statement

The authors declare that the research was conducted in the absence of any commercial or financial relationships that could be construed as a potential conflict of interest.

## Supplementary material

Supplementary Figure S1 Flow cytometry analysis of Ly6G+/CD11b+ double-positive neutrophils after isolation. Bone marrow-derived neutrophils were isolated using a Percoll gradient fractionation system consisting of a three-layer Percoll gradient (52, 64, and 72%) and neutrophils were isolated from the 64 to 72% interface, washed, and used in this assay. The cells were washed with PBS, blocked using 1% BSA, labeled with anti-Ly6G and anti-CD11b specific antibodies and analyzed by flow cytometry. Two representative isolates from wild type (WT) and *Nlrp3*^A350V^ mice are shown.

Supplementary Figure S2 The uninduced Nlrp3^A350V^ MWS mice show normal levels of granule markers in plasma. The plasma levels of azurophilic granule cargo myeloperoxidase (MPO) **(A)** and gelatinase granule cargo (MMP-9) **(B)** were analyzed by ELISA in uninduced (pre-Tam) or tamoxifen-induced (post-Tam) MWS (*Nlrp3* mutant) mice as described under “Materials and Methods.” Mean ± SEM, (*n* = 3). Unpaired Student's *t*-test.

Supplementary Figure S3 Expression levels of Nlrp3 protein in wild type and Nlrp3^*A350V*^ MWS mice. Total protein extracts from spleens from 3 wild type and 3 *Nlrp3*^*A350V*^ *MWS* mice were resolved by gel electrophoresis, transferred to nitrocellulose and analyzed by Western blot. **(A)** Immunoblot analysisi of NLRP3 expression in three control and three *Nlrp3*^*A350V*^ *MWS* spleens are shown. **(B)** Equal loading was determined by Ponceau S staining. **(C)** Quantitative analysis of normalized protein expression, mean ± SEM, (*n* = 3).
