# Supplementary figures and images for: Increased Neutrophil Secretion Induced by NLRP3 Mutation Links the Inflammasome to Azurophilic Granule Exocytosis
Source: Front Cell Infect Microbiol. 2017 Dec 11;7:507. doi: 10.3389/fcimb.2017.00507 (PMC5732154; doi:10.3389/fcimb.2017.00507)

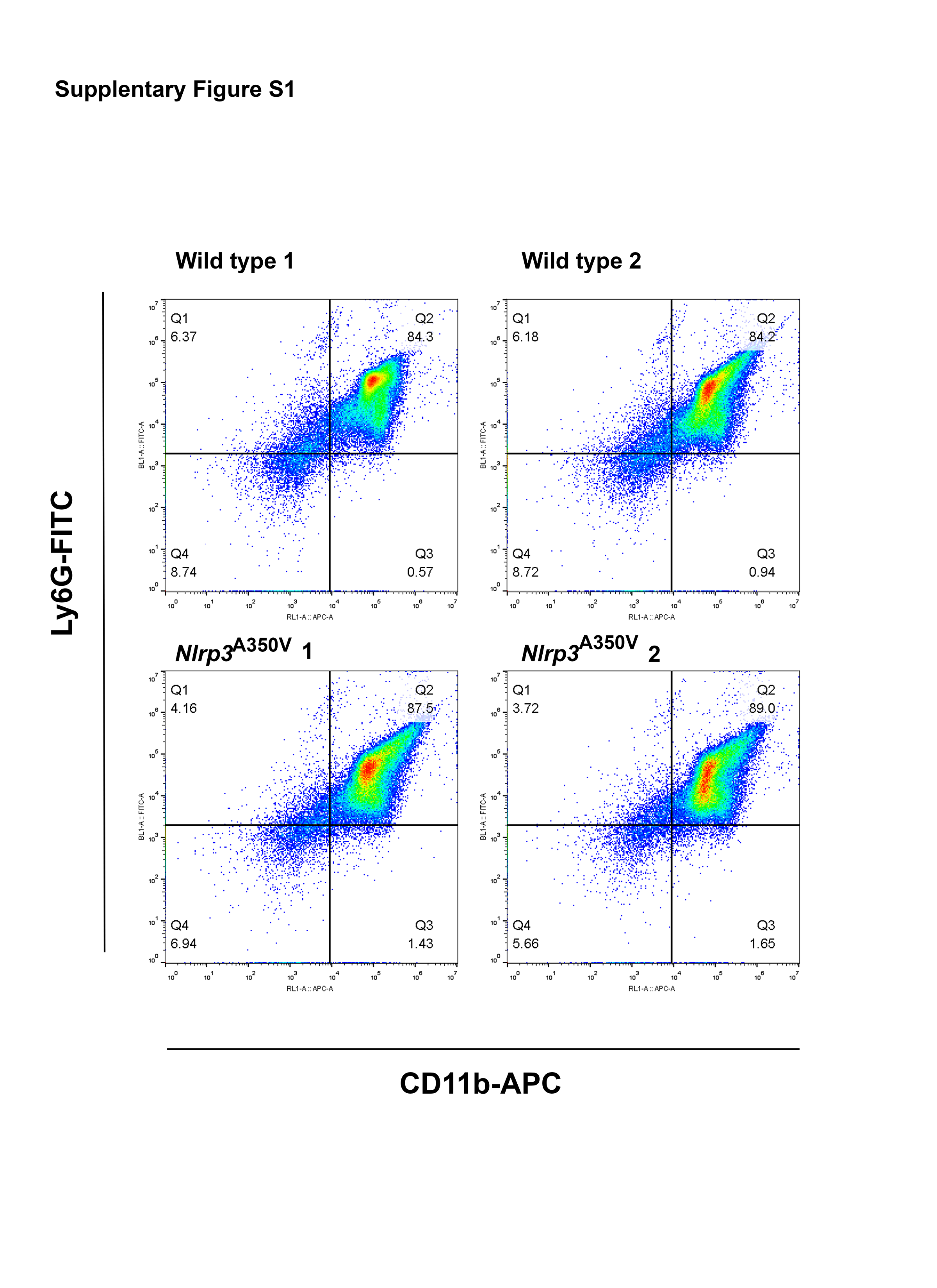

Supplement: Supplementary Figure S1 — Flow cytometry analysis of Ly6G+/CD11b+ double-positive neutrophils after isolation. Bone marrow-derived neutrophils were isolated using a Percoll gradient fractionation system consisting of a three-layer Percoll gradient (52, 64, and 72%) and neutrophils were isolated from the 64 to 72% interface, washed, and used in this assay. The cells were washed with PBS, blocked using 1% BSA, labeled with anti-Ly6G and anti-CD11b specific antibodies and analyzed by flow cytometry. Two representative isolates from wild type (WT) and Nlrp3A350V mice are shown. [file Image1.TIF]

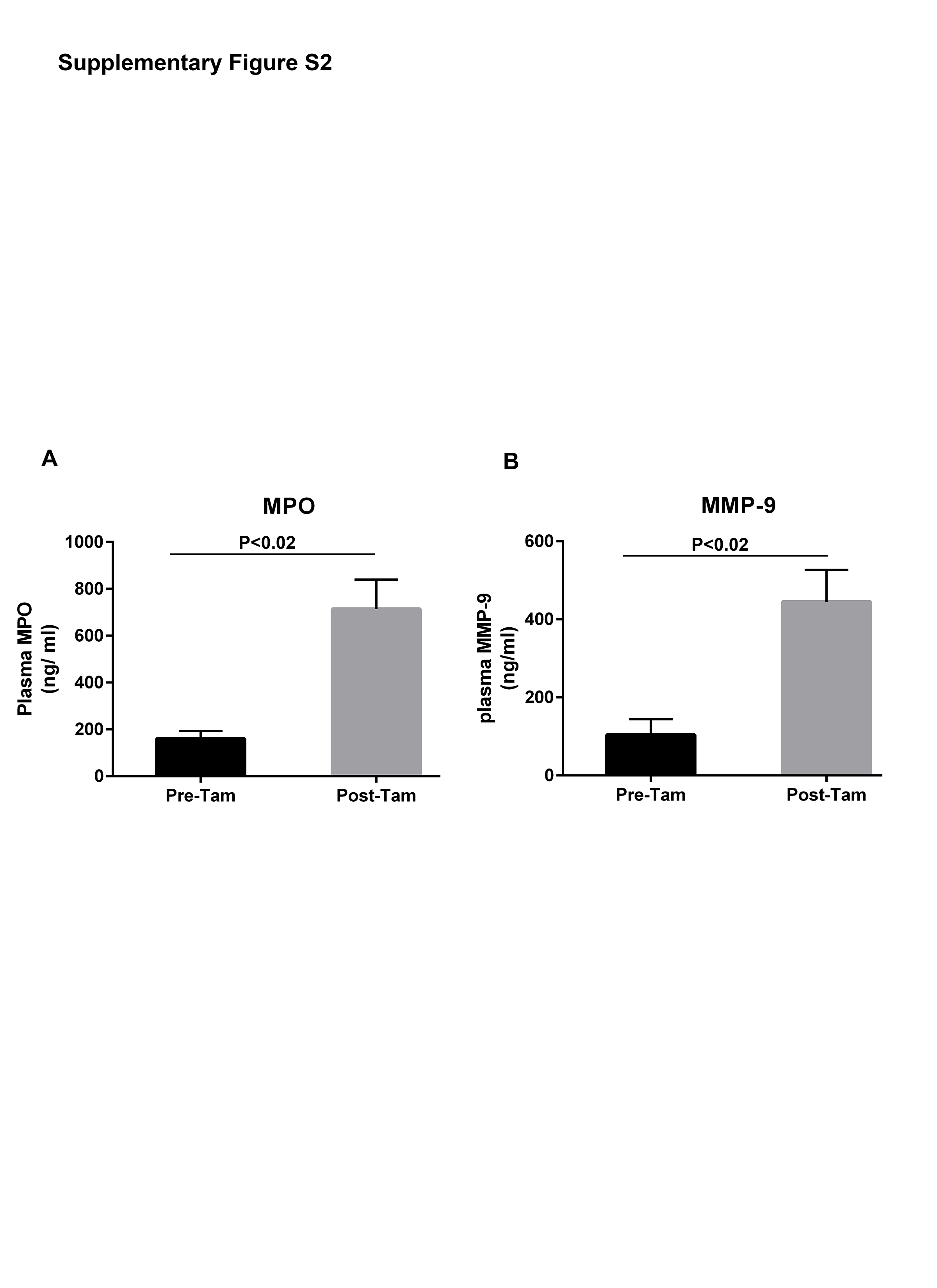

Supplement: Supplementary Figure S2 — The uninduced Nlrp3A350V MWS mice show normal levels of granule markers in plasma. The plasma levels of azurophilic granule cargo myeloperoxidase (MPO) (A) and gelatinase granule cargo (MMP-9) (B) were analyzed by ELISA in uninduced (pre-Tam) or tamoxifen-induced (post-Tam) MWS (Nlrp3 mutant) mice as described under “Materials and Methods.” Mean ± SEM, (n = 3). Unpaired Student's t-test. [file Image2.TIF]

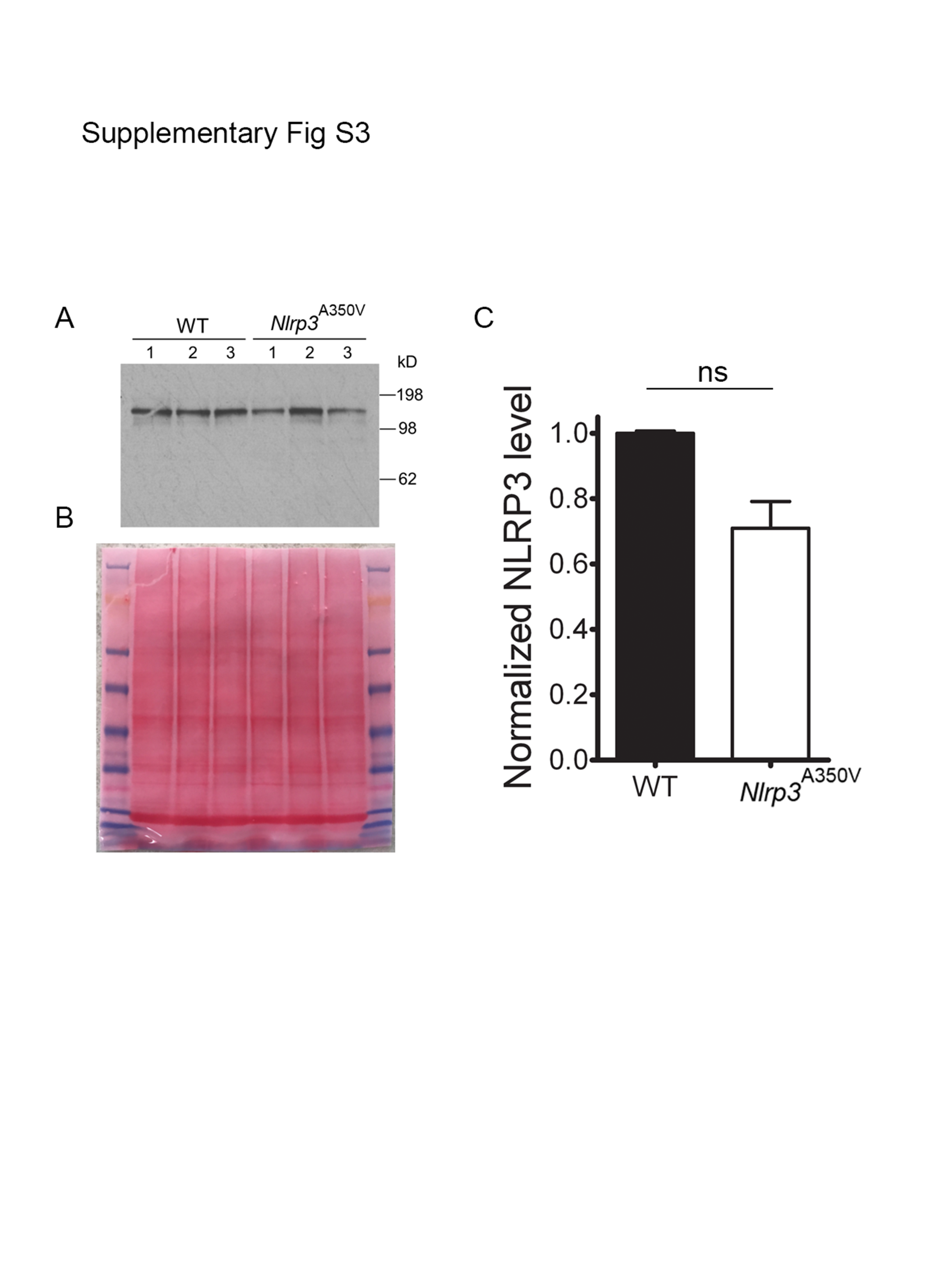

Supplement: Supplementary Figure S3 — Expression levels of Nlrp3 protein in wild type and Nlrp3A350V MWS mice. Total protein extracts from spleens from 3 wild type and 3 Nlrp3A350V MWS mice were resolved by gel electrophoresis, transferred to nitrocellulose and analyzed by Western blot. (A) Immunoblot analysisi of NLRP3 expression in three control and three Nlrp3A350V MWS spleens are shown. (B) Equal loading was determined by Ponceau S staining. (C) Quantitative analysis of normalized protein expression, mean ± SEM, (n = 3). [file Image3.TIF]
